# LncRNA SNHG20 promotes cell proliferation and invasion by suppressing miR-217 in ovarian cancer

**DOI:** 10.1007/s13258-021-01138-4

**Published:** 2021-07-24

**Authors:** Xuefeng Xing, Ming An, Tonghua Chen

**Affiliations:** Sanya People’s Hospital, Sanya, Hainan China

**Keywords:** Ovarian cancer, LncRNA SNHG20, MiR-217, Proliferation, Invasion

## Abstract

**Background:**

Ovarian cancer is the most common female gynecological malignancy. SNHG20, as a long non-coding RNA, has been proven to be an important regulator in the occurrence and development of various tumors. However, the potential mechanism of SNHG20 in ovarian cancer is unclear.

**Objective:**

The present study was aimed to investigate the functions and mechanisms of SNHG20 in ovarian cancer.

**Methods:**

The expression of SNHG20 and miR-217 in ovarian cancer tissues and cell lines was detected by qRT-PCR. CCK-8 assay was used to measure cell proliferation in transfected cells. The transwell assay was used to detect the relative invasion rate of transfected cells. The putative binding sites between SNHG20 and miR-217 were predicted by software LncBase v.2, and the interaction between SNHG20 and miR-217 was confirmed by dual-luciferase reporter assays and RIP assay. The rescue experiments were used to illustrate potential mechanisms.

**Results:**

SNHG20 was upregulated in ovarian cancer tissues and cell lines. Overexpression of SNHG20 promoted ovarian cancer cell proliferation and invasion. MiR-217 was downregulated in ovarian cancer tissues and cells, and was negatively regulated by SNHG20. Moreover, miR-217 overexpression inhibited ovarian cancer cell proliferation and invasion. Furthermore, miR-217 mimic reversed the inhibitory effect of SNHG20 overexpression on the biological behavior of ovarian cancer cells.

**Conclusions:**

SNHG20 promoted cell proliferation and invasion by sponging miR-217 in ovarian cancer. These results suggested that SNHG20 and miR-217 might provide new targets for therapeutic application in ovarian cancer.

**Supplementary Information:**

The online version contains supplementary material available at 10.1007/s13258-021-01138-4.

## Introduction

Ovarian cancer is one of the most common female gynecologic malignancy and is the fifth leading cause of cancer-related death in women (Webb et al. 2017; Siegel et al. 2017). The overall prognosis of patients with ovarian cancer has improved due to dramatic improvements in diagnosis and therapeutic approaches (Holmes 2015). However, most patients with ovarian cancer are already in the advanced stage when they are diagnosed (Chien and Poole 2017). Advanced ovarian cancer is prone to drug resistance, recurrence, and metastasis, which leads to a 5-year survival rate of less than 40% (Allemani et al. 2015; Matz et al. 2017; Sankaranarayanan and Ferlay 2006). Thus, it is necessary to further explore the molecular mechanisms of the occurrence and development of ovarian cancer and to develop new treatment strategies.

A growing number of studies suggested that non-coding protein sequences in the genome were involved in the occurrence and metastasis of cancer, and were expected to become cancer therapeutic targets (Panoutsopoulou et al. 2018; Yoshida and Kimura 2017). Long non-coding RNA (lncRNA) is a type of non-coding RNA longer than 200 nucleotides, which plays an important regulatory role in the occurrence and development of tumors (Bill et al. 2019; Huarte 2015). However, the functions of lncRNA in tumors are complex, and many of them are unknown. Currently, the lncRNA-microRNAs (miRNAs) interaction network is often used as a research direction (Wang et al. 2015). Previous researches have indicated that some lncRNAs were dysregulated in ovarian cancer and played vital roles in tumorigenesis and progression, such as MAIT (Zhou et al. 2020), PVT1 (Chen et al. 2018), HOTAIR (Chang et al. 2018), and SNHG17 (Pan et al. 2020). The long non-coding RNA small nucleolar RNA host gene 20 (SNHG20) is located on 17q25.2, which has been reported to act as an oncogene in various tumors (Zhao et al. 2019), such as prostate cancer (Wu et al. 2019), gastric cancer (Yu et al. 2019), bladder cancer (Zhao et al. 2018), and lung cancer (Chen et al. 2017). Further researches have shown that SNHG20 knockdown inhibited ovarian cancer cell proliferation, migration, invasion, epithelial-mesenchymal transition and promoted cell apoptosis (He et al. 2018; Wang et al. 2019). However, the potential molecular mechanism of SNHG20 in ovarian cancer is unclear.

MicroRNA (miRNA) is a short non-coding RNA with 18–22 nucleotides in length (Tristán-Ramos et al. 2020), which always regulates gene expression through targeting mRNAs. Mounting evidence suggested that MiR-217 functions as a tumor suppressor. It has been reported that miR-217 could suppress cell proliferation, migration, and invasion in non-small cell lung cancer by regulating SIRT1 and P53/KAI1 signaling (Jiang et al. 2020). In gastric cancer, miR-217 inhibited epithelial-to-mesenchymal transition through targeting PTPN14 (Chen et al. 2020). A study revealed that miR-217 suppressed the migration and invasion of HeLa cells through modulating MAPK1 (Zhu et al. 2019). In ovarian cancer, miR-217 acted as a tumor suppressor role by targeting IGF1R (Li et al. 2016). However, further studies on the role of miR-217 in ovarian cancer are needed, and the link between SNHG20 and miR-217 is still unclear.

LncRNA can interact with miRNA and act as a competitive endogenous RNA (ceRNA) to regulate the expression of target genes, thus playing an important role in the occurrence and development of tumors (Bartel 2009). In this study, we aimed to explore the expression pattern, role, and potential functional mechanism of SNHG20 in ovarian cancer. Then, we further studied the relationship between SNHG20 and miR-217 in the proliferation and invasion of ovarian cancer cells. We also hope to find novel theoretical targets for the treatment of ovarian cancer.

## Materials and methods

### Clinical sample selection

A total of 30 surgically resected ovarian cancer tissues and adjacent normal tissues were collected at the Sanya People’s Hospital from 2017 to 2019. The characteristics of 30 patients with ovarian cancer in this study are presented in Table S1. All clinical samples were immediately snap-frozen in liquid nitrogen and then stored at 80 °C until RNA isolation. Besides, the research was approved by the Ethics Committee of the Sanya People’s Hospital (Approval No. 2017. 138).

### Cell culture

Ovarian cancer cell lines (SKOV3 and A2780) and normal human ovarian surface epithelial cell line (HOSEpiC) were obtained from American Type Culture Collection (ATCC, Manassas, VA, USA). All cells were cultured in RPMI-1640 medium (Gibco, Thermo Fisher Scientific) supplemented with 10% fetal bovine serum (FBS, Gibco; Thermo Fisher Scientific), and incubated in a humidified atmosphere at 37 °C with 5% CO_2_.

### Cell transfection

SNHG20 small interference RNA (si-SNHG20) or its negative control (si-NC), miR-217 mimics and inhibitor (miR-217 and anti-miR-217) or its control (miR-NC and anti-miR-217) were synthesized by GenePharma Co., Ltd (Shanghai, China). The overexpression vector pcDNA 3.1 was acquired from Thermo Fisher Scientific. The above oligonucleotides and SNHG20 overexpression plasmid (pcDNA-SNHG20) or its negative control (pcDNA) were transfected into ovarian cancer cell lines (SKOV3 and A2780) using Lipofectamine™ 3000 transfection reagent (Invitrogen, Carlsbad, CA, USA), according to the manufacturer’s suggested protocols.

### Total RNA extraction and quantitative real-time polymerase chain reaction (qRT-PCR)

Total RNA was extracted from human ovarian cancer tissues and cell lines using Trizol reagent (Invitrogen, Thermo Fisher Scientific, Inc.). Reverse transcription was performed to acquire complementary DNA using the PrimeScript RT reagent Kit (Takara, Tokyo, Japan). Real-time PCR was performed using SYBR^®^ Premix Ex Taq™ II Kit (Takara, Tokyo, Japan) by an ABI 7500 real-time PCR system (Applied Biosystem, MA, USA). All primers were designed and synthesized by Sangon Biotech Co., Ltd. (Sangon, Shanghai, China). GAPDH (glyceraldehyde-3-phosphate dehydrogenase) and U6 were used as internal controls for SNHG20 and miR-217, respectively. The primer sequences were listed as follows: SNHG20 forward, 5′-ATGGCTATAAATAGATACACGC-3′ and reverse, 5′-GGTACAAACAGGGAGGGA-3′; miR-217 forward, 5′-CGGCTACTGCATCAGGAACTG-3′ and reverse, 5′-CGGCCCAGTGTTCAGACTAC-3′; GAPDH forward, 5′-GACCACAGTCCATGCCATCAC-3′ and reverse, 5′-ACGCCTGCTTCACCACCTT-3′; U6 forward, 5′-CGCTTCGGCAGCACATATACTA-3′ and reverse, 5′-ATGGAACGCTTCACGAATTTGC-3′. The 2^−ΔΔCt^ method was used to analyze the expression of SNHG20 or miR-217.

### Cell counting kit-8 (CCK-8) assay

The proliferation of ovarian cancer cells was measured by cell counting kit-8 (CCK-8, Beyotime Institute of Biotechnology, Shanghai, China). SKOV3 or A2780 cells (4 × 10^3^ per well) with different transfection were cultured in 96-well plants. After 0, 24, 48, or 72 h of incubation, 10 µl of CCK-8 solution was added into each well, then the cells were incubated at 37 °C for 2 h. The absorbance at 450 nm of transfected cells was measured with a microplate reader (Thermo Fisher, USA).

### Transwell assay

Cell invasion ability was assessed by transwell chambers (10 μm pore size; BD Biosciences, San Jose, CA, USA). SKOV3 or A2780 cells (1 × 10^5^ cells per well) were cultured in the upper chamber, and the upper chamber was pre-coated with 100 µl of Matrigel gel (BD, Franklin Lakes, NJ, USA). At the same time, RPMI-1640 medium supplemented with 10% FBS was added to the lower chamber. After incubation for 24 h, the cells on the lower surface were fixed with methanol. At last, the wells were stained with 0.5% crystal violet solution and observed by a microscope.

### Dual-luciferase reporter assay

The SNHG20 sequences containing the binding sites (SNHG20-WT) and non-binding sites (SNHG20-MUT) for miR-217 were synthesized and inserted into a psiCHECK2 reporter vector (Promega, WI, USA). The SNHG20-WT or SNHG20-MUT reporter vector was co-transfected into SKOV3 and A2780 cells with anti-miR-217 (miR-217 inhibitor) or anti-NC (inhibitor NC), then the cells were seeded into 24-well plates. After transfection for 48 h, the luciferase activity was assessed by the Dual-Luciferase Reporter Assay Kit (Promega, WI, USA).

### RNA immunoprecipitation (RIP) assay

The Magna RIP RNA Binding Protein Immunoprecipitation Kits (Millipore, Billerica, MA, USA) were used for the RIP assays. The operation steps of RIP analysis were consistent with the previously mentioned description (Ma et al. 2020).

### Statistical analysis

GraphPad Prism 8. (GraphPad Software, Inc.) and SPSS 22.0 (SPSS Inc., Chicago, IL, USA) were used for data analysis. All experiments were carried out in three independent biological replicates, and the data were expressed as the mean ± SD (standard deviation). The difference between the two groups was compared by the paired or unpaired Student’s t-test. The relationship between SNHG20 expression and miR-217 was analyzed by Spearman’s correlation coefficient. *P* < 0.05 was considered statistically significant.

## Results

### SNHG20 was up-regulated in ovarian cancer tissues and cell lines

**Fig. 1:**
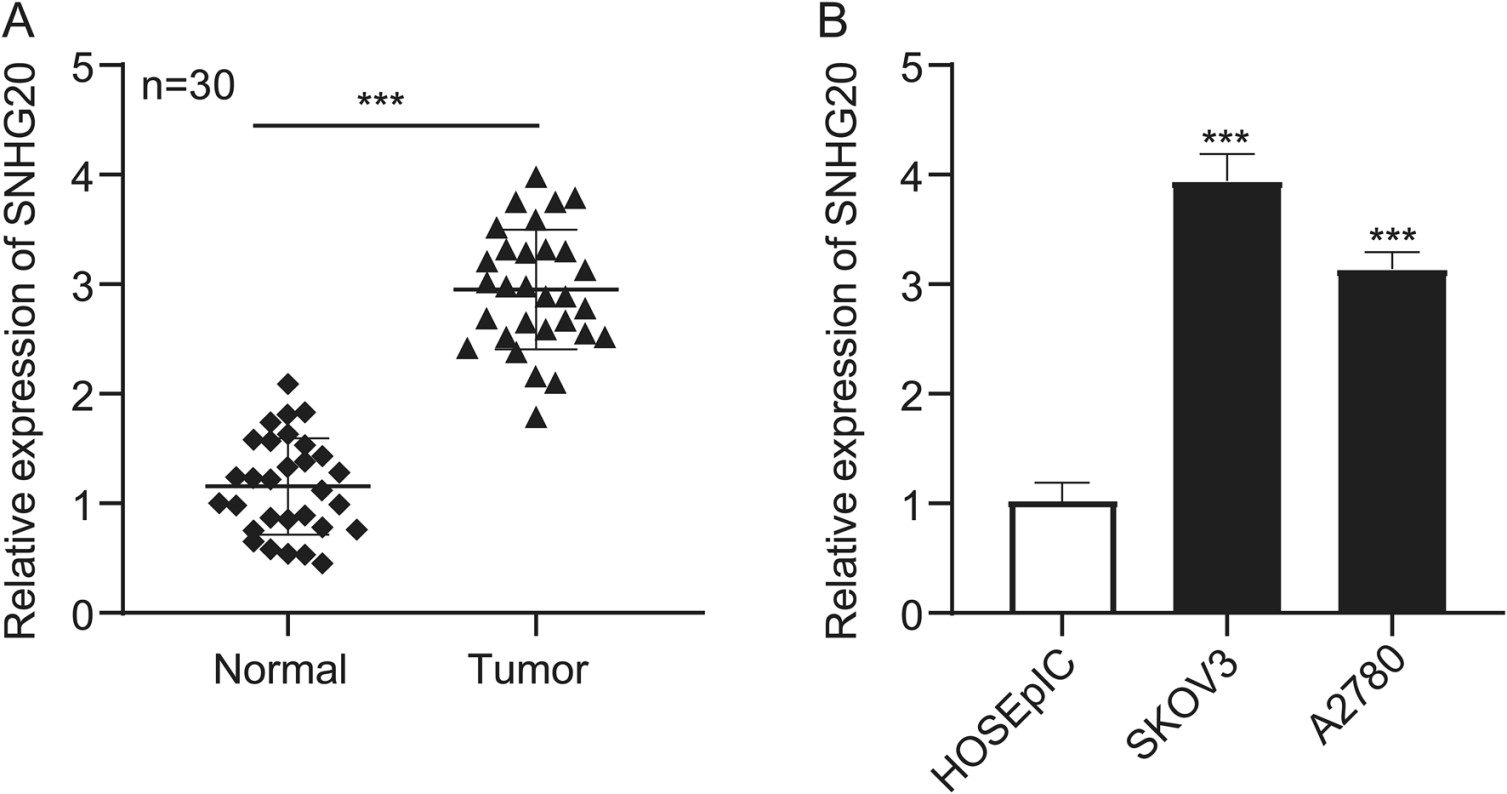
SNHG20 was significantly overexpressed in ovarian cancer tissues and cell lines. **a** QRT-PCR analysis of SNHG20 expression in ovarian cancer tissues (n = 30) and adjacent normal tissues (n = 30). **b** The SNHG20 expression in ovarian cancer cell lines and normal human ovarian surface epithelial cell line (HOSEpiC). **P < 0.01

First, the expression of SNHG20 in 30 ovarian cancer tissues and their adjacent normal tissues was measured by qRT-PCR assay. The result showed that SNHG20 was overexpressed in ovarian cancer compared with that in adjacent normal tissues (Fig. 1a). In addition, we investigated whether there were some relationships between SNHG20 expression and ovarian cancer stages. The qRT-PCR data suggested that there were no significant differences in SNHG20 expression associated with different tumor stages (Fig. S1). Then, SNHG20 expression in ovarian cancer cell lines (SKOV3 and A2780) and human ovarian surface epithelial cell line (HOSEpiC) were also determined by qRT-PCR assay. The qRT-PCR data showed that SNHG20 expression was significantly higher in ovarian cancer cell lines (SKOV3 and A2780) than in HOSEpiC cell line (Fig. 1b). These results indicated that dysregulated SNHG20 might involve in the occurrence and development of ovarian cancer.

### SNHG20 positively promoted ovarian cancer cell proliferation and invasion in vitro

**Fig. 2:**
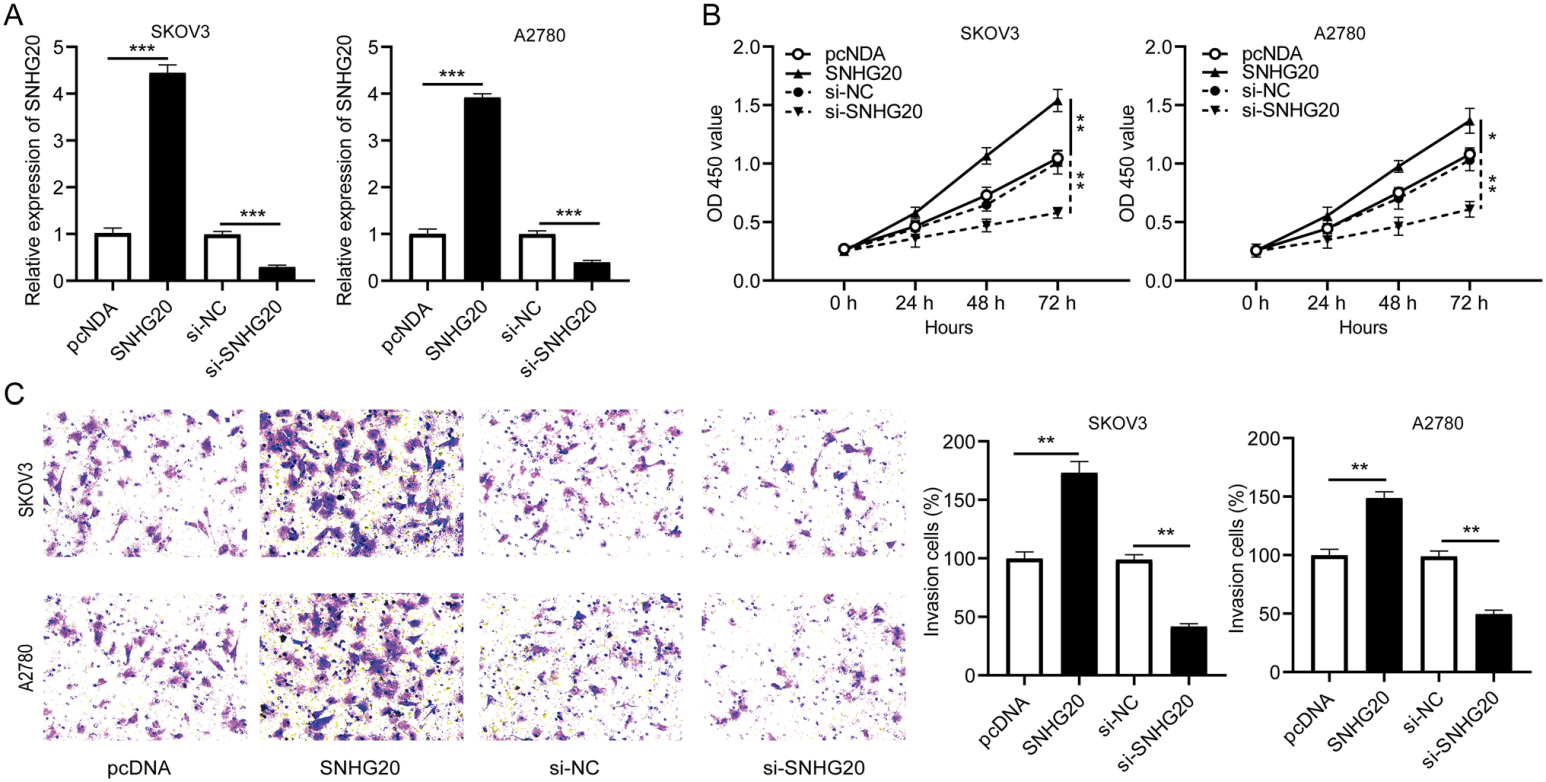
Effects of lncRNA SNHG20 on proliferation and invasion of ovarian cancer cells. SKOV3 and A2780 cells were transfected with pcDNA, SNHG20, si-NC, or si-SNHG20, respectively. pcDNA and si-NC were used as controls. **a** Expression of SNHG20 in transfected cells was measured by qRT-PCR. **b** Proliferation ability of transfected cells was assessed by CCK-8 assay. **c** The transwell assay was used to assess the relative invasion rate of transfected cells. **P < 0.01, ***P < 0.001

To explore the functional role of SNHG20 in ovarian cancer, SKOV3 and A2780 cells were transfected with pcDNA, SNHG20, si-NC, or si-SNHG20. First, the endogenous expression level of SNHG20 was measured. The qRT-PCR results showed that SNHG20 expression was remarkably increased in SKOV3 and A2780 cells transfected with SNHG20, and the expression of SNHG20 was significantly decreased after transfection of si-SNHG20 (Fig. 2a). Next, the CCK-8 assay was performed to measure the ability of cell proliferation. The data indicated that SNHG20 significantly promoted cell proliferation and SNHG20 knockdown inhibited cell proliferation (Fig. 2b). Besides, transwell assay concluded that the number of invasive cells was collectively enhanced in SKOV3 and A2780 cells transfected with SNHG20, and knockdown of SNHG20 weakened cell invasion (Fig. 2c). In short, these data indicated that SNHG20 regulated ovarian cancer cell proliferation and invasion.

### miR-217 was a target of SNHG20

**Fig. 3:**
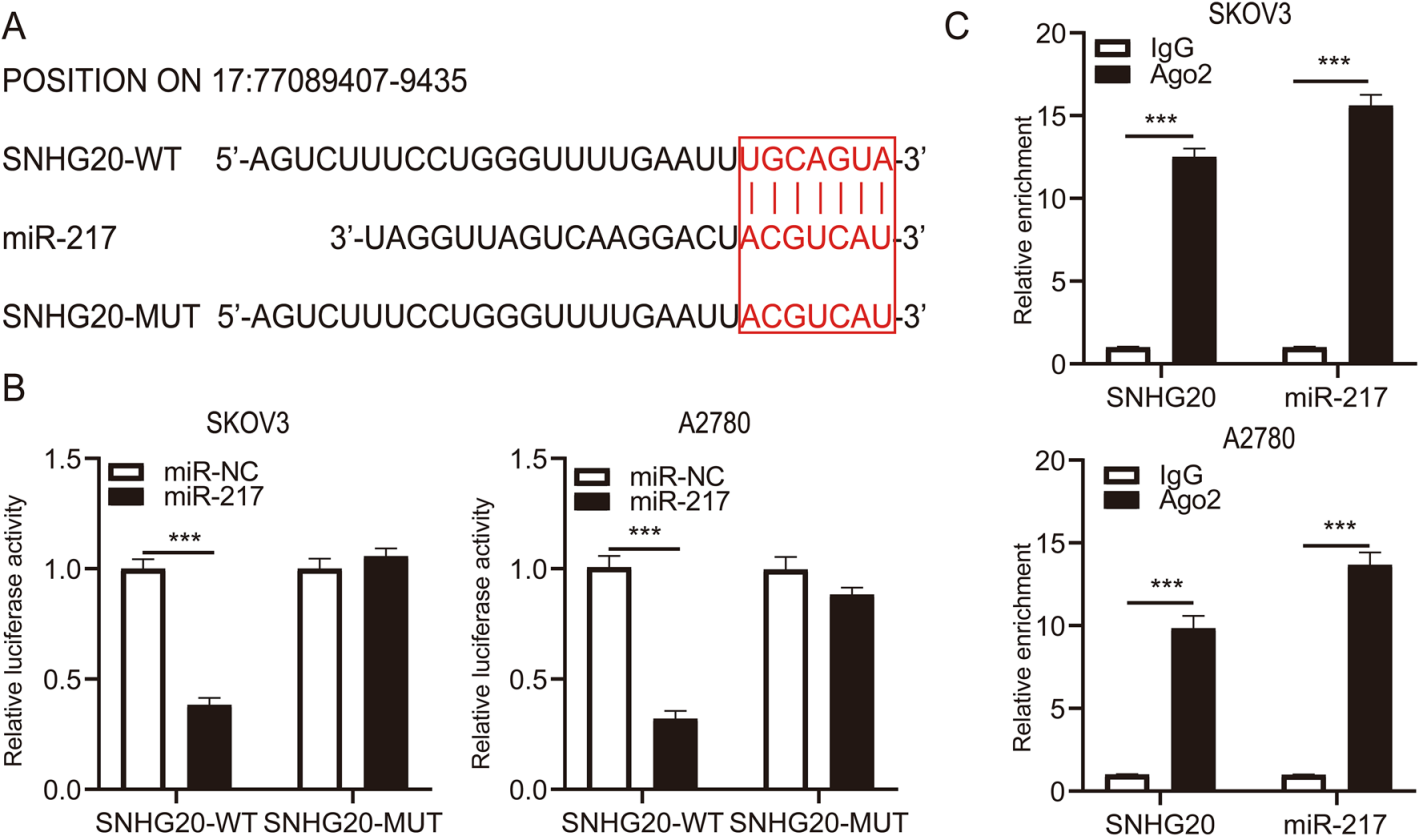
SNHG20 directly interacted with miR-217. **a** The putative binding sites between SNHG20 and miR-217 were analyzed by software LncBase v.2. **b** The relationship between SNHG20 and miR-217 was confirmed by dual-luciferase reporter assay. **c** The interaction between SNHG20 and miR-217 was further confirmed by RIP assay. ***P < 0.001

To further study the molecular mechanism of SNHG20 in ovarian cancer, the potential targets of SNHG20 were predicted using the online software LncBase Predicted v.2. The results showed that miR-217 was a target gene of SNHG20, and the binding sites between SNHG20 and miR-217 were shown in Fig. 3a. Then, to confirm whether there was direct interaction, the luciferase reporter vector was co-transfected into SKOV3 and A2780 cells with miR-217 or miR-NC. The luciferase activity of SKOV3 and A2780 cells in the SNHG20-WT group was markedly reduced by introducing miR-217, whereas the mutant vector was unaffected (Fig. 3b). Furthermore, the RIP assay indicated that SNHG20 and miR-217 were significantly enriched in human Ago2 antibodies compared with the IgG antibody control (Fig. 3c). All the above data implied that miR-217 was binding SNHG20.

### miR-217 expression was reduced in ovarian cancer tissues and SKOV3 and A2780 cells, and it was regulated by SNHG20

**Fig. 4:**
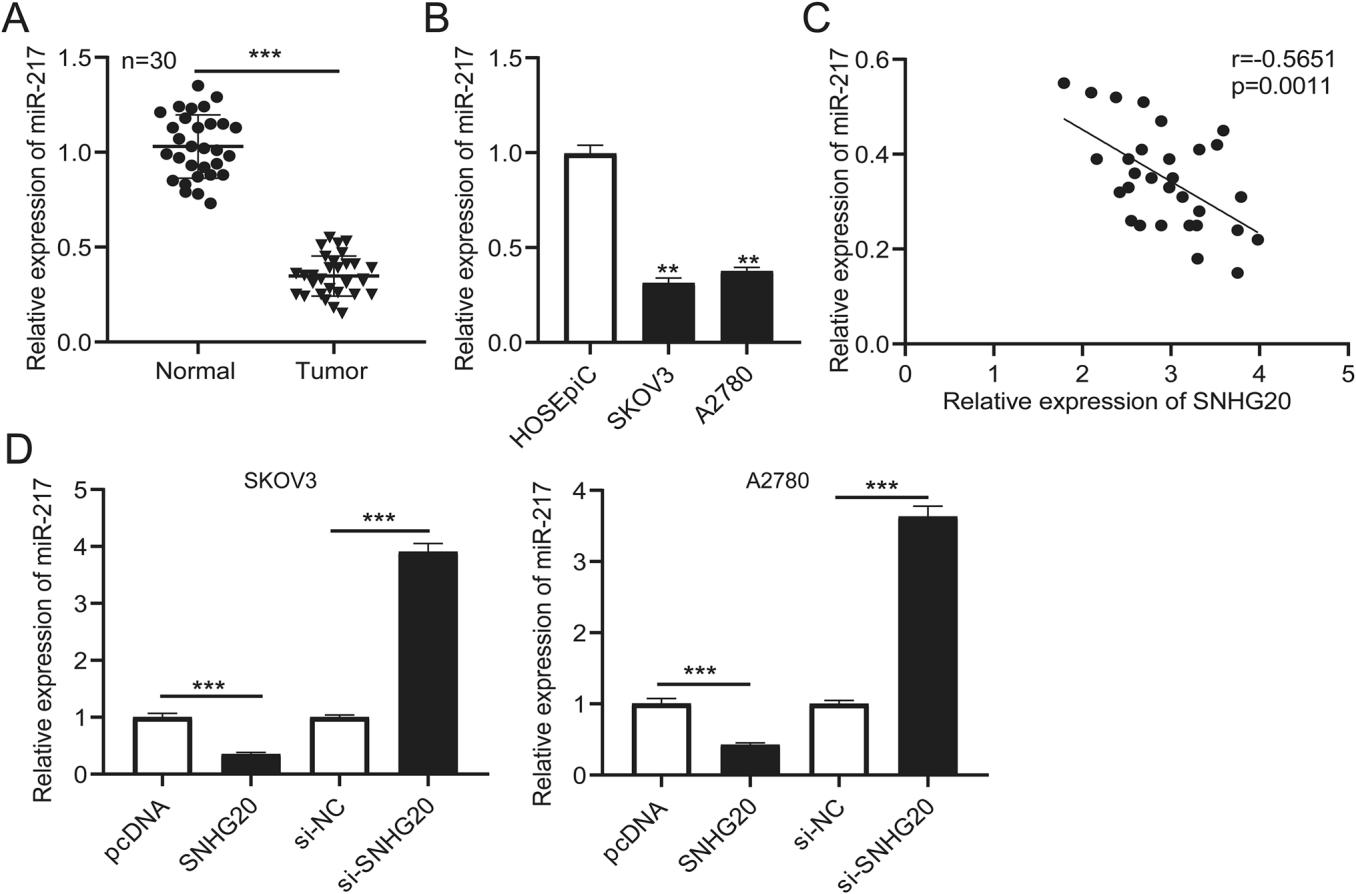
Expression of miR-217 was declined in ovarian cancer tissues and cell lines and was directly regulated by SNHG20. **a** Expression of miR-217 in ovarian cancer tissues and normal tissues was measured by qRT-PCR. **b** The expression of miR-217 in ovarian cancer cells and HOSEpiC cells. **c** Correlation analysis between SNHG20 expression level and miR-217 expression level in ovarian cancer tissues. **d** Expression of miR-217 was measured by qRT-PCR in SKOV3 and A2780 cells transfected with pcDNA, SNHG20, si-NC, or si-SNHG20. **P < 0.01, ***P < 0.001

Next, the expression of miR-217 in ovarian cancer tissues and SKOV3 and A2780 cells was measured by qRT-PCR assay. The qRT-PCR data suggested that the expression of miR-217 in ovarian cancer and SKOV3 and A2780 cells were lower than in normal tissues and cells (Fig. 4a, b). The correlation between SNHG20 and miR217 expression was performed by Pearson’s correlation analysis. The results indicated that the expression of miR-217 was negatively correlated with SNHG20 in ovarian cancer tissues (Fig. 4c). Furthermore, the SKOV3 and A2780 cells were transfected with pcDNA, SNHG20, si-NC, or si-SNHG20, and the expression of miR-217 in these cells was examined. The results determined that miR-217 expression was significantly reduced in SKOV3 and A2780 cells transfected with SNHG20 and were remarkably increased with a low SNHG20 expression (Fig. 4d). These results demonstrated that miR-217 might act as a potential role in ovarian cancer, and the expression of miR-217 was negatively regulated by SNHG20.

### Overexpression of miR-217 suppressed ovarian cancer cell proliferation and invasion

**Fig. 5:**
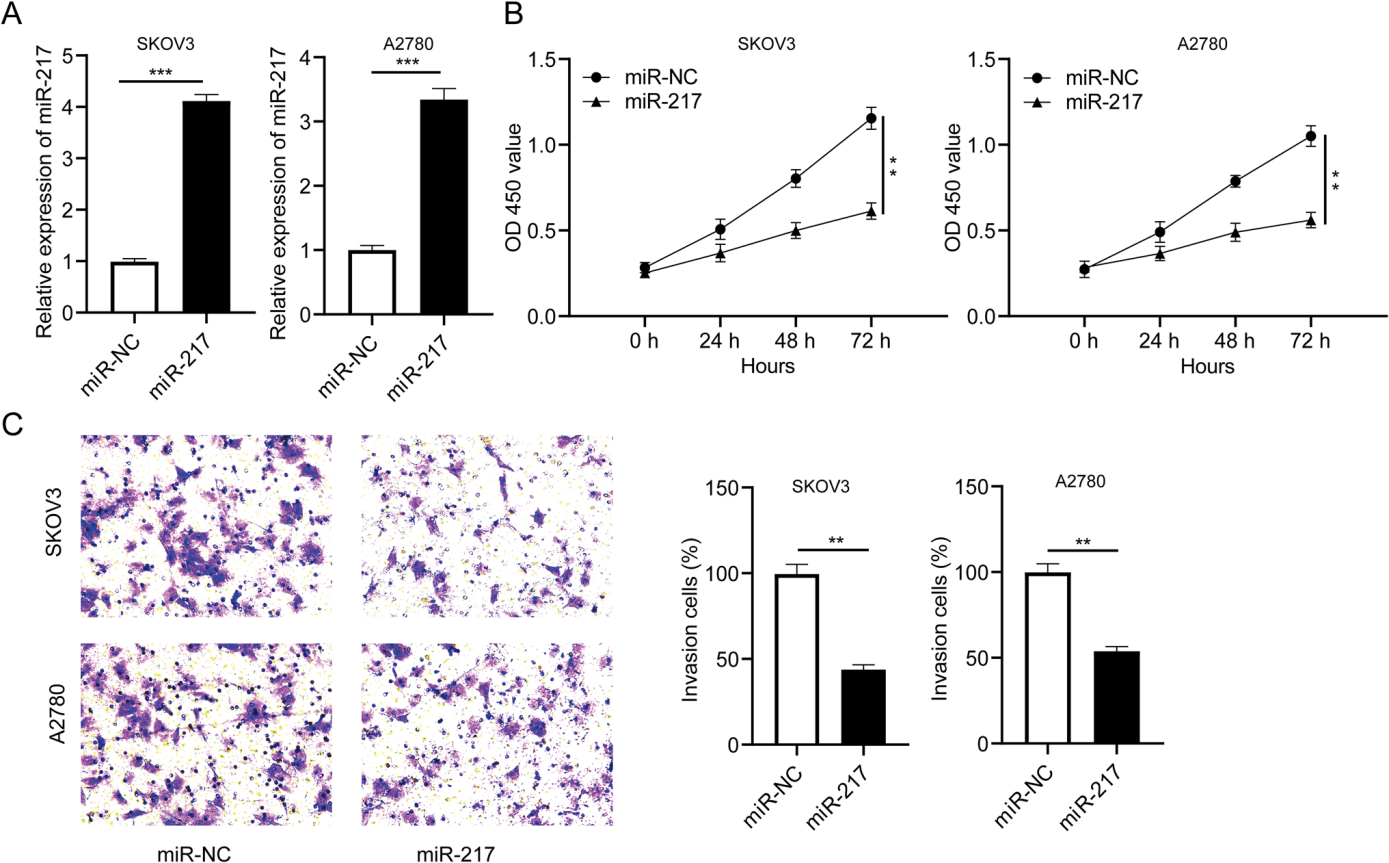
MiR-217 inhibited cell proliferation and invasion in ovarian cancer. SKOV3 and A2780 cells were transfected with miR-217 mimic, and miR-NC was used as control. **a** Expression of miR-217 in transfected cells was checked by qRT-PCR assay. **b** The proliferation ability of transfected cells was assessed by CCK-8 assay. **c** The relative invasion rate of transfected cells was evaluated by transwell assay. **P < 0.01, ***P < 0.001

To explore the effect of miR-217 on ovarian cancer cell growth and invasion, SKOV3 and A2780 cells were transfected with miR-NC or miR-217 mimic, respectively. Compared with the control group, the miR-217 expression was remarkably increased in ovarian cancer cells SKOV3 and A2780 transfected with miR-217 (Fig. 5a), while there was no significant difference in SNHG20 expression (Fig. S2). Moreover, the CCK-8 assay indicated that miR-217 overexpression suppressed cell proliferation (Fig. 5b). Furthermore, the transwell assay confirmed that overexpression of miR-217 reduced the ability of cell invasion (Fig. 5c). These results suggested that miR-217 might play a negative role in the proliferation and invasion of ovarian cancer cells.

### SNHG20 promoted cell proliferation and invasion by inhibiting miR-217 expression

**Fig. 6:**
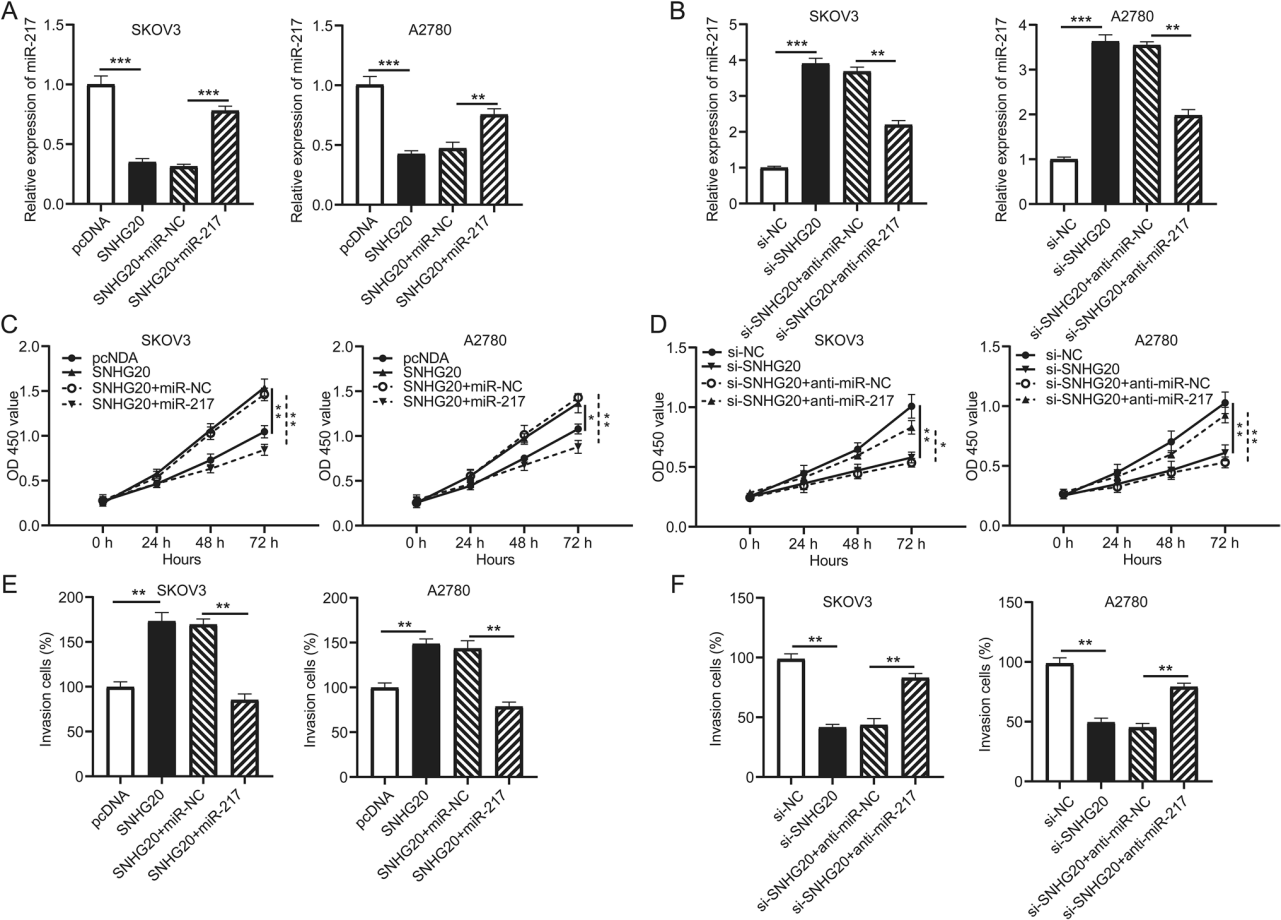
SNHG20 promoted cell proliferation and invasion by regulating miR-217 in ovarian cancer. SKOV3 and A2780 cells were transfected with pcDNA, SNHG20, SNHG20 + miR-NC, SNHG20 + miR-217, si-NC, si-SNHG20, si-SNHG20 + anti-miR-NC, and si-SNHG20 + anti-miR-217, respectively. **a**, **b** Expression of miR-217 in transfected cells was determined by qRT-PCR assay. **c**, **d** The CCK-8 assay was used to assess the proliferation ability of transfected cells. **e**, **f** The transwell assay was used to detect the relative invasion rate of transfected cells

To clarify the relationship between SNHG20 and miR-217 clearly, some rescue experiments were designed in vitro. The qRT-PCR data exhibited that miR-217 mimic reversed the inhibition of endogenous miR-217 expression in SKOV3 and A2780 cells transfected with SNHG20 (Fig. 6a), and miR-217 inhibitor (anti-miR-217) reversed the promotion of miR-217 expression in SKOV3 and A2780 cells transfected with si-SNHG20 (Fig. 6b). Additionally, miR-217 suppressed cell proliferation in SKOV3 and A2780 cells transfected with SNHG20 (Fig. 6c), and anti-miR-217 promoted cell proliferation in ovarian cancer cells transfected with si-SNHG20 (Fig. 6d). Moreover, the ability of cell invasion was decreased in SKOV3 and A2780 cells co-transfected with SNHG20 + miR-217 (Fig. 6e), and si-SNHG20 + anti-miR-217 co-transfection was increased (Fig. 6f). These results implied that SNHG20 regulated cell proliferation and invasion via suppressing miR-217 expression in ovarian cancer.

## Discussion

Ovarian cancer is a common gynecological cancer. Most patients with ovarian cancer are diagnosed at an advanced stage due to the lack of specific clinical manifestations of early ovarian cancer (Chien and Poole 2017). The low 5-year survival rate was also responsible for the higher mortality rate of ovarian cancer (Li et al. 2018). Recently, increasing studies on the molecular mechanism of ovarian cancer carcinogenesis have been reported, and many molecular mechanisms are still unknown. Hence, more efforts are needed to reveal the underlying molecular mechanism of ovarian cancer. Moreover, accumulated studies have shown that lncRNAs played an important role in tumorigenesis, and acted as potential treatment targets in cancer. In this study, we clarified the functions and molecular mechanism of lncRNA SNHG20 in ovarian cancer.

SNHG20, a member of small nucleolar RNA host genes, has been verified to make a big difference in tumorigenesis. A previous study has shown that SNHG20 was markedly elevated in glioma cells, and inhibition of SNHG20 expression could increase the apoptosis of glioma cells (Guo et al. 2019). Jin et al. reported that SNHG20 was overexpressed in non-small cell lung cancer, and knockdown of SNHG20 inhibited proliferation, migration and invasion of non-small cell lung cancer and increased apoptosis (Jin et al. 2019). It was proved that SHNG20 expression was significantly increased in prostate cancer, and SNHG20 overexpression promoted prostate cancer cell proliferation and invasion and decreased cell apoptosis (Wu et al. 2019). In ovarian cancer, SNHG20 promoted ovarian cancer progression via Wnt/β-catenin signaling (He et al. 2018) or by regulating the proliferation regulators and epithelial-mesenchymal transition (EMT)-related proteins (Wang et al. 2019). Consistent with previous studies, we verified that the expression of SNHG20 was upregulated in ovarian cancer tissues and SKOV3 and A2780 cells. Knockdown of SNHG20 inhibited the proliferation and invasion of ovarian cancer cells, whereas SNHG20 overexpression promoted cell growth and invasion. Taken together, SNHG20 might play an important role in the cell proliferation and invasion of ovarian cancer.

In recent years, it has been confirmed that miRNAs regulated cell growth, invasion, metastasis, and apoptosis in cancer (Cao et al. 2020; Yang et al. 2020). Many miRNAs have been determined to be involved in the development of ovarian cancer. MiR-134-3p inhibited the progression of ovarian cancer via targeting flap structure-specific endonuclease 1 in vitro (Zhao et al. 2020). MiR-195 regulated tumor growth and MICU1 expression in ovarian. MiR-6086 suppressed ovarian cancer angiogenesis via regulating the OC2/VEGFA/EGFL6 axis (Wu et al. 2020). Besides, overexpression of miR-217 in epithelial ovarian cancer cells inhibited cell proliferation, migration, and invasion (Li et al. 2016). In the present study, our findings indicated that miR-217 was decreased in ovarian cancer tissues and cell lines, and miR-217 overexpression inhibited the proliferation and invasion in vitro. The above results suggested that miR-217 was involved in the proliferation and invasion of ovarian cancer as a tumor suppressor.

Previous studies have confirmed that lncRNAs are widely involved in the network of ceRNA regulation and can act as ceRNA and further function as miRNA sponge to regulate the transcription level of miRNA (Xin et al. 2018). To further investigate the potential mechanism of SNHG20 in ovarian cancer, the online software LncBase v.2 was used to predict the target genes of SNGH20. The results showed that miR-217 was a target gene of SNHG20. Then, the Pearson’s correlation analysis indicated that the expression of miR-217 was negatively correlated with SNHG20 in ovarian cancer tissues. Besides, the direct interaction of SNHG20 with miR-217 was confirmed by dual-luciferase reporter gene and RIP analysis, and miR-217 expression was regulated by SNHG20 in vitro. Furthermore, overexpression of miR-217 suppressed the proliferation and invasion and reversed the effect of SNHG20 in ovarian cancer cells. All these results proved that SNHG20 promoted ovarian cancer cell proliferation and invasion by inhibiting miR-217 expression. The previous study has shown that miR-217 played an inhibitory role in epithelial ovarian cancer by suppressing IGF1R expression (Li et al. 2016). However, it was unknown whether SNHG20/miR-217 regulated the progression of ovarian cancer by regulating IGF1R gene or other target genes. Therefore, exploring the downstream genes of SNHG20/miR-217 in ovarian cancer is the focus of our future work. In addition, future investigations are needed to determine more specific functions of SNHG20/miR217 in ovarian cancer cells, such as their role in cell apoptosis.

In conclusion, lncRNA SNHG20 was up-regulated in ovarian cancer tissues and cell lines. Up-regulated SNHG20 promoted growth and invasion of ovarian cancer cells, while knockdown of SNHG20 inhibited cell proliferation and invasion. Software predictive analysis indicated that miR-217 was a target gene of SNHG20. Further research showed that miR-217 expression was negatively regulated by SNHG20. Besides, miR-217 could reverse the effect of SNHG20 overexpression. Together, SNHG20 promoted the proliferation and invasion of ovarian cancer via suppressing miR-217.

## Supplementary Information

Below is the link to the electronic supplementary material.

Supplementary file1 (DOCX 13 KB)

Supplementary file2 (TIF 1052 KB)

Supplementary file3 (TIF 1051 KB)

## Data Availability

The data in this research is available from the corresponding author on reasonable request.
