# Supplementary material for: LncRNA SNHG20 promotes cell proliferation and invasion by suppressing miR-217 in ovarian cancer
Source: Genes Genomics. 2021 Jul 24;43(9):1095–104. doi: 10.1007/s13258-021-01138-4 (PMC8376724; doi:10.1007/s13258-021-01138-4)
Supplement: Supplementary file 1 — Supplementary file1 (DOCX 13 KB) [file 13258_2021_1138_MOESM1_ESM.docx]

**Table S1** The characteristics of 30 patients with ovarian cancer.

| **Variable** | **Group** | **Case (n, %)** |
| --- | --- | --- |
| Age (years) | ≥50  <50 | 21 (70%)  9 (30%) |
| FIGO stage | I  II  III  IV | 6 (20%)  12 (40%)  7 (23.3%)  5 (16.7%) |
| Histological grade | G1-2  G3 | 18 (60%)  12 (40%) |
| Tumor size (cm) | ≥2  <2 | 16 (53.3%)  14 (46.7%) |
| Familial status | sporadic  familial | 23 (76.7%)  7 (23.3%) |
| Lymph node metastasis | Negative  Positive | 21 (70%)  9 (30%) |
| CA-125 (U/mL) | ≥500  <500 | 10 (33.3%)  20 (66.6%) |
