# Supplementary figures and images for: LncRNA SNHG20 promotes cell proliferation and invasion by suppressing miR-217 in ovarian cancer
Source: Genes Genomics. 2021 Jul 24;43(9):1095–104. doi: 10.1007/s13258-021-01138-4 (PMC8376724; doi:10.1007/s13258-021-01138-4)

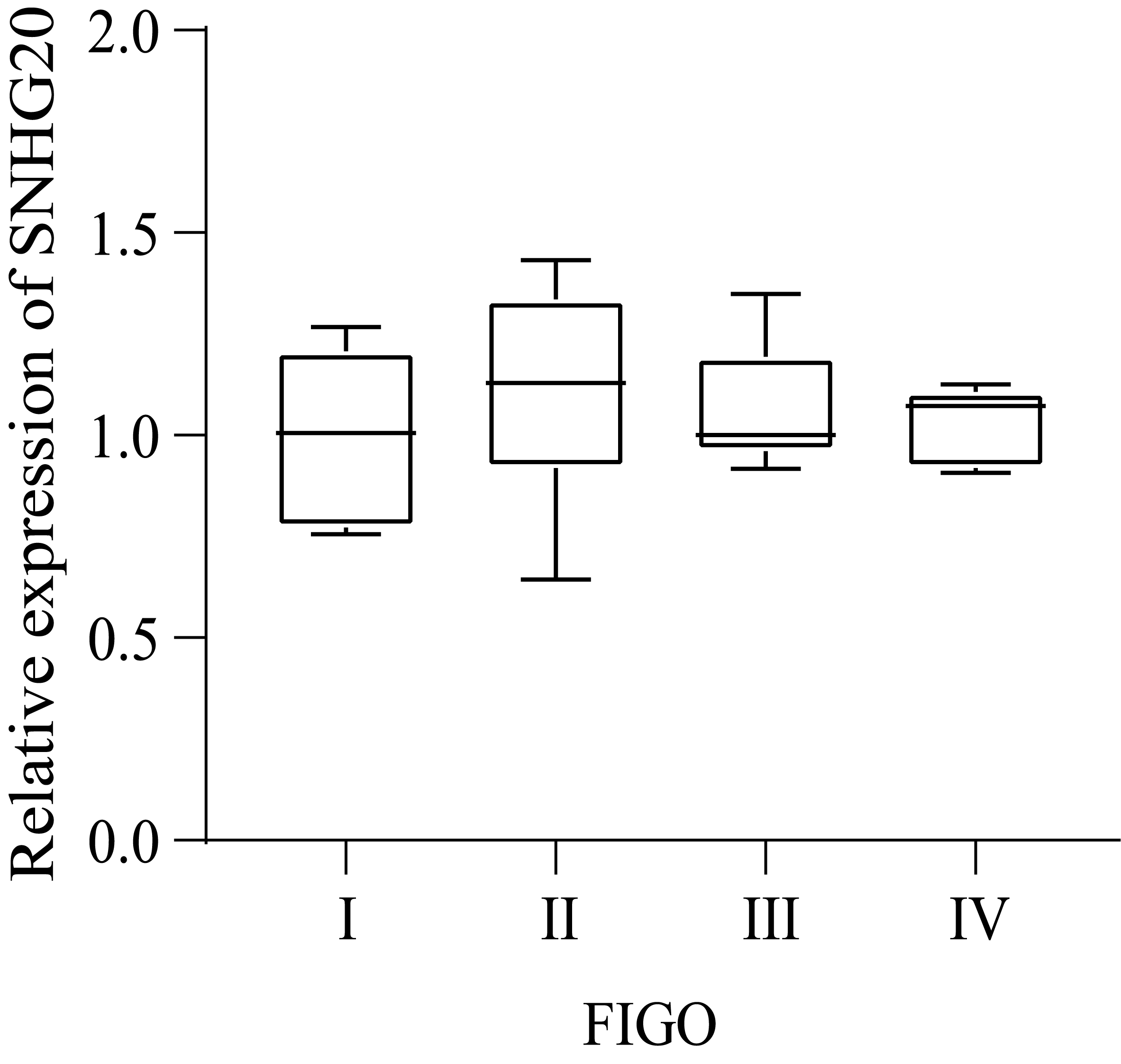

Supplement: Supplementary file 2 — Supplementary file2 (TIF 1052 KB) [file 13258_2021_1138_MOESM2_ESM.tif]

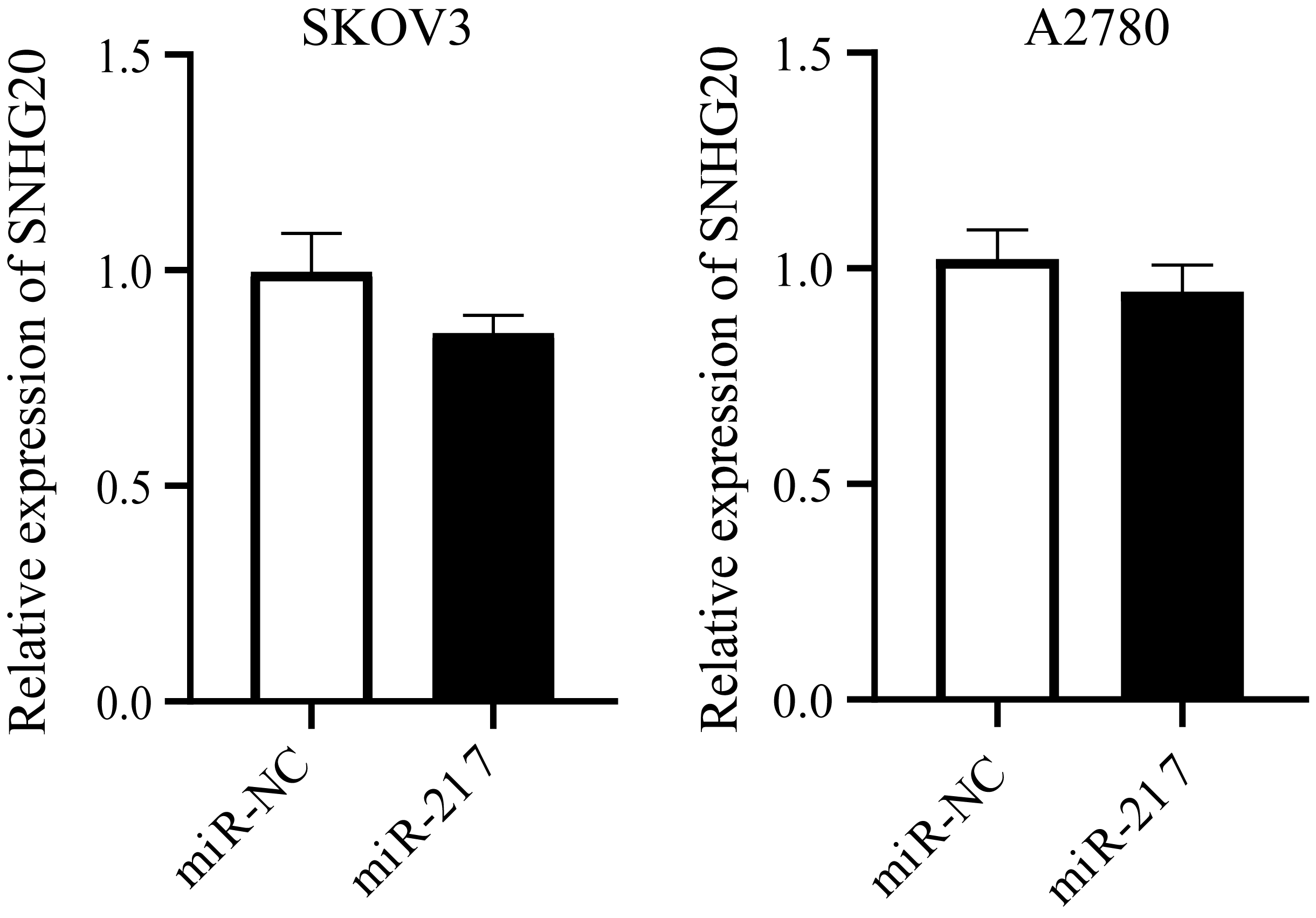

Supplement: Supplementary file 3 — Supplementary file3 (TIF 1051 KB) [file 13258_2021_1138_MOESM3_ESM.tif]
